# Development of a Genetic Risk Score to predict the risk of overweight and obesity in European adolescents from the HELENA study

**DOI:** 10.1038/s41598-021-82712-4

**Published:** 2021-02-04

**Authors:** Miguel Seral-Cortes, Sergio Sabroso-Lasa, Pilar De Miguel-Etayo, Marcela Gonzalez-Gross, Eva Gesteiro, Cristina Molina-Hidalgo, Stefaan De Henauw, Frederic Gottrand, Christina Mavrogianni, Yannis Manios, Maria Plada, Kurt Widhalm, Anthony Kafatos, Éva Erhardt, Aline Meirhaeghe, Diego Salazar-Tortosa, Jonatan Ruiz, Luis A. Moreno, Luis Mariano Esteban, Idoia Labayen

**Affiliations:** 1 Growth, Exercise, NUtrition and Development (GENUD) Research Group, Instituto Agroalimentario de Aragón (IA2), Instituto de Investigación Sanitaria Aragón (IIS Aragón), Universidad de Zaragoza, Zaragoza, Spain; 2 Spanish National Cancer Research Centre (CNIO), Madrid, Spain; 3 Instituto de Investigación Sanitaria Aragón (IIS Aragón), Zaragoza, Spain; 4 Centro de Investigación Biomédica en Red de Fisiopatología de la Obesidad y la Nutrición (CIBERObn), Instituto de Salud Carlos III, Madrid, Spain; 5 Institute of Nutritional and Food Sciences, Nutritional Physiology, University of Bonn, Bonn, Germany; 6 ImFine Research Group, Department of Health and Human Performance, Facultad de Ciencias de la Actividad Física y del Deporte-INEF, Universidad Politécnica de Madrid, Madrid, Spain; 7 EFFECTS 262 Department of Medical Physiology, School of Medicine, University of Granada, 18071 Granada, Spain; 8 Department of Public Health, Faculty of Medicine and Health Sciences, Ghent University, Ghent, Belgium; 9 Faculty of Medicine, University Lille, Lille, France; 10 Department of Nutrition and Dietetics, School of Health Science and Education, Harokopio University, Athens, Greece; 11 University of Crete School of Medicine, Crete, Greece; 12 Division of Gastroenterology and Hepatology, Department of Internal Medicine III, Medical University of Vienna, Austria and Austrian Academic Institute for Clinical Nutrition, Vienna, Austria; 13 Faculty of Medicine, University of Crete, Crete, Greece; 14 Department of Pediatrics, Medical School, University of Pécs, Pecs, Hungary; 15 UMR1167, RID-AGE, Risk Factors and Molecular Determinants of Aging-Related Diseases, Centre Hosp. Univ Lille, Institut Pasteur de Lille, Université de Lille, Lille, France; 16 Department of Ecology and Evolutionary Biology, University of Arizona, Tucson, AZ USA; 17 Departmento de Actividad Física y Deporte, Faculty of Sport Sciences, Universidad de Granada, Granada, Spain; 18 Escuela Politécnica de La Almunia, Universidad de Zaragoza, Zaragoza, Spain; 19 Department of Health Sciences, Public University of Navarra, Pamplona, Spain

**Keywords:** Risk factors, Genetics, Genetic markers, Genomics

## Abstract

Obesity is the result of interactions between genes and environmental factors. Since monogenic etiology is only known in some obesity-related genes, a genetic risk score (GRS) could be useful to determine the genetic predisposition to obesity. Therefore, the aim of our study was to build a GRS able to predict genetic predisposition to overweight and obesity in European adolescents. A total of 1069 adolescents (51.3% female), aged 11–19 years participating in the Healthy Lifestyle in Europe by Nutrition in Adolescence (HELENA) cross-sectional study were genotyped. The sample was divided in non-overweight (non-OW) and overweight/obesity (OW/OB). From 611 single nucleotide polymorphisms (SNP) available, a first screening of 104 SNPs univariately associated with obesity (*p* < 0.20) was established selecting 21 significant SNPs (*p* < 0.05) in the multivariate model. Unweighted GRS (uGRS) was calculated by summing the number of risk alleles and weighted GRS (wGRS) by multiplying the risk alleles to each estimated coefficient. The area under curve (AUC) was calculated in uGRS (0.723) and wGRS (0.734) using tenfold internal cross-validation. Both uGRS and wGRS were significantly associated with body mass index (BMI) (*p* < .001). Both GRSs could potentially be considered as useful genetic tools to evaluate individual’s predisposition to overweight/obesity in European adolescents.

## Introduction

Childhood obesity is a major public health problem^1^. Pediatric obesity increases the risk of physical and psychological health problems already in childhood, and later in adulthood^2^. More so, adiposity related disorders predominantly diagnosed in adults such as type 2 diabetes mellitus (T2DM) and cardiovascular diseases might originate in early life, and potentially reduce life expectancy. Over the last two decades, large-scale studies have been unveiling new common variants in locus of certain genes related with childhood and adult obesity^3–5^. At least 97 loci have been associated with obesity^6^. Currently, the *FTO* gene still remains the locus explaining the largest association with obesity in adults, children and adolescents^7,8^. In this regard, previous studies have shown that each copy of the *FTO* rs9939609 polymorphism A allele is associated with 2.8% higher body fat in European adolescents^9,10^. Some studies have found some associations between single nucleotide polymorphisms (SNPs) with obesity risk factors, being potentially useful as early life risk indicators in children and adults^11^. However, individual SNPs can explain little of disease variance^12^. Several studies have demonstrated the potential value of other genetic approaches that combine a number of SNPs to develop a genetic risk score (GRS) by summing the number of risk alleles: unweighted GRS (uGRS) or by multiplying the number of risk alleles to each estimated coefficient: weighted GRS (wGRS)^13–15^. The creation and validation of obesity-specific GRS sets a landmark in personalised genetic risk prediction for obesity and obesity-related diseases^16^. Different obesity-related GRS have been constructed in adults^15–18^ and children^19,20^ with significant obesity-gene associations, being implemented on a variety of ethnic population backgrounds. Within European populations, Seyednasrollah et al.^21^ computed two weighted GRS (wGRS) of 97 and 19 SNPs previously related to the risk of obesity in two cohorts including 2262 Finnish children and adolescents (3–18 years). Further, Viljakainen et al.^22^ developed a wGRS to predict the risk of overweight and obesity in a cohort of 1142 Finnish preadolescents (11.3 ± 0.2 years) considering body mass index (BMI) and 30 BMI-related SNPs from previous genome-wide association studies (GWAS). As only few studies testing obesity risk in European adolescents with GRSs have been conducted, the aim of the present study was to develop a GRS for overweight and obesity in adolescents participating in the Healthy Lifestyle in Europe by Nutrition in Adolescence (HELENA) cross-sectional study.

## Methods

### Study design and population

The data were extracted from the HELENA multicentric and cross-sectional study containing a total sample of 4356 adolescents (51.6% females), aged 11–19 years old, from 10 European cities located in separated geographical points in Europe in 2006–2007. Their size of the cities was large enough to ensure participants diversity^23^. The main objective of the HELENA study was to obtain comparable data of a large sample of European adolescents on nutrition and health-related parameters by a standardised procedure^24^. More so, the study was performed following the ethical guidelines of the Declaration of Helsinki 1964 (revision of 2013), the Good Clinical Practice, and the legislation about clinical research in humans in each of the participating countries and was approved by the Ethics Committee of each city participating in the study^25^. The protocol was approved by the Ethical Committee (Comité de Ética de la Investigación de la Comunidad Autónoma de Aragón: CEICA). Written informed consent and assent to participate in the study were obtained from adolescents and their parents before being enrolled. One third of the subjects (N = 1172) from the total sample were randomly selected for blood sampling^24^. After including specific inclusion criteria from genomic parameters (SNPs) and anthropometry (BMI), a total of 1069 adolescents (51.3% females) were finally considered for the analysis in the present study. The flow chart of the selected sample is displayed in Supplementary Fig. 1.

**Figure 1:**
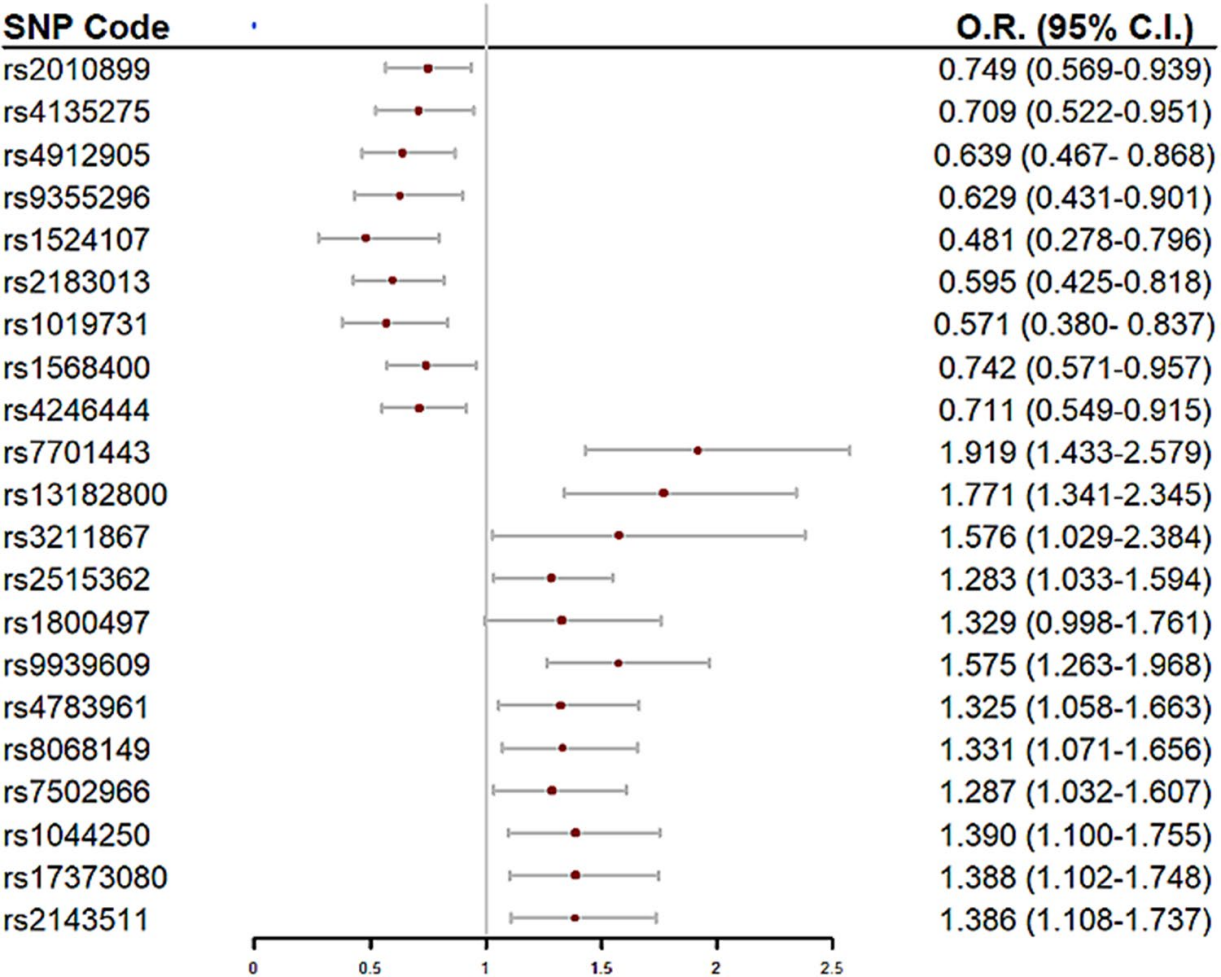
Forest plot of single nucleotide polymorphisms (SNPs) negatively (OR < 1) and positively (OR > 1) associated with risk of OW/OB. Legend: SNPs ordered by chromosome number. Protective SNPs against risk of overweight/obesity are shown in the upper part of the forest plot; SNPs with predisposition risk to overweight/obesity are shown in the bottom part. Multivariate model Odds Ratio (O.R.) and 95% confidence intervals (C.I.) displayed.

### Physical examination

All measurements were performed by trained researchers following standard protocols. Weight and height were measured following standard procedures^26^. BMI was calculated from height and weight (kg/m^2^)^27^ and categorised into non-overweight (non-OW) and overweight, including obesity (OW/OB), according to the age- and sex-specific BMI international cut-offs proposed by the World Obesity Federation^28^. FMI was calculated was calculated dividing fat mass (FM) by height squared (in meters).

### Blood collection and genotyping

The blood samples were collected in overnight fasting state. A standardised methodology for blood collection, transport and analysis was performed by a certified laboratory^29^. Blood for DNA extraction was collected in EDTA K3 tubes and stored at the Institute of Nutritional and Food Sciences (IEL) of the University of Bonn, and sent to the Laboratoire d'Analyse Genomique Centre de Ressources Biologiques (LAG-CRB) e BB- 0033-00071 Institut Pasteur de Lille, F-59000 Lille, France. DNA was extracted from white blood cells with the Puregene kit (QIAGEN, Courtaboeuf, France) and stored at − 20 °C. The genotyping was done by an Illumina system (Illumina, Inc, San Diego, California) using the Golden- Gate technology (Sampling procedure scheme, GoldenGate; Software, Inc, San Francisco, California). In terms of gene selection within the HELENA study, a candidate gene approach was used. First, a certain number of behaviour and metabolic pathways related to the adolescence´s health were identified. These included included food intake, eating behaviour, food choices and preferences, energy and adipose tissue metabolism, glucose, insulin, lipid and lipoprotein metabolism among others. Finally, SNPs playing a key role in genes coding for the abovementioned pathways were selected. The HapMap database was used to select tag and independent SNPs. SNPs were selected with a minor allele frequency (MAF) above 0.1 and tag SNPs with r^2^ above 0.8. If tag SNPs described for a single gene exceeded in number (more than ~ 20), only SNPs significantly associated with appropriate phenotypes in previous studies were selected, if available. Lastly, SNPs from the NCBI database were used when a limited number of SNPs were available in the HapMap database.

### Statistical analysis

Descriptive characteristics by sex are shown as median and interquartile range (IQR) for continuous variables and as absolute and relative frequency for categorical ones. The statistical tests used to compare differences by sex were Pearson´s chi-square for categorical variables and Mann–Whitney-Wilcoxon test for continuous variables. Pearson’s chi-square statistic test was used to analyse the Hardy–Weinberg equilibrium. Shapiro–Wilk non-parametric test to check normality of variables was performed.

All statistical analyses were performed using RStudio Version 1.2.5001 (*RStudio Team (2015). RStudio: Integrated Development for R. RStudio, Inc., Boston, MA URL* http://www.rstudio.com/*)* and the significance level was set at *p* < 0.05.

### Development of the genetic risk score

Candidate gene approach was the procedure based to select the genes in the HELENA study. First, relevant adolescent´s behaviours and metabolic pathways related to health were identified. Second, key proteins that, according to the literature, play a role in these pathways were also identified. Third, a selection of SNPs coding for these proteins was performed. Fourth, in order to select and tag SNPs independently, the HapMap database (2007 release) was used. SNPs with a minor allele frequency (MAF) above 0.1 and tag SNPs with r^2^ above 0.8 were selected. If too many tag SNPs described for a single gene (more than ~ 20) were identified, only SNPs significantly associated with appropriate phenotypes in previous publications were selected, if available. Finally, SNPs from the NCBI database were included when a too limited number of SNPs were available in the HapMap database. Based on the above, a total of 611 SNPs related to obesity and obesity-related phenotypes available in the HELENA dataset^30^ were used to build a GRS considering BMI as obesity-related variable in order to predict a major predisposition of overweight/obesity in European adolescents^24^. Each SNP was recoded as 0, 1, or 2 depending on the number of risk alleles defined in previous literature, respectively. A further selection of SNPs was performed using generalised linear model (GLM) to establish an initial cut off point (*p* < 0.20) to refine the search to 104 SNPs. Then, a step by step algorithm was applied to select the significant SNPs under the *p* < 0.05 threshold in a multivariate model to shortlist a final number of 21 SNPs significantly associated with BMI. The correspondence between actual and predicted probabilities of this model was analysed by a calibration curve. The unweighted GRS (uGRS) was calculated by summing the number of risk alleles from the 21 SNP variants with a rescaling, considering the SNPs that appear as protector factors. The wGRS was the result of multiplying the number of risk alleles at each locus (0, 1, 2) for each estimated coefficient of the multivariate model. Participants with missing data were dismissed in the GRS analysis (N = 3287). Receiver operating characteristics (ROC) curve analysis^31^ was applied to test the diagnostic accuracy of the GRS to classify potential participants for obesity associated disturbances^32^. The area under curve (AUC) was calculated in uGRS and wGRS considering weight status as binary variable (i.e., non-OW vs. OW/OB). Selection of uGRS over wGRS to proceed with the design of the final model was performed by the higher value of the AUC compared using the Delong test. The model was internally validated performing tenfold cross validation analysis. For this analysis, the whole dataset was divided in 10 groups, using 9 of them to build the predictive model and the one to remains to validate this model. This procedure was repeated taking into account all possible ways to select the 9 subgroups, ensuring different forms to validate GRS with data not used in the building model process. Moreover, we evaluated the distribution of uGRS and wGRS values for NON-OW and OW/OB in a boxplot to graphically analyse the performance of the GRS. In order to provide the best cut-off for the use of the GRS as a dichotomic variable, the maximisation of the Youden index^33^ was explored (see Table 3). Lastly, to test the GRS reliability with a general adiposity estimate other than BMI, simple linear regression models (LRM) were performed to evaluate the association between fat mass index (FMI) and both wGRS and uGRS.

### Informed consent

Informed consent was signed by parents of all participants. The datasets used and/or analysed during the current study are available from the corresponding author on reasonable request.

## Results

### Description of the study sample: demographics

The sample was composed of 520 boys and 549 girls. Main characteristics of study participants are shown in Supplementary Table 1. The median age of participants barely differed between males (14.6 years, IQR: 13.6—15. 7) and females (14.6 years, IQR: 13.5–15.7) (*p* = 0.722). The prevalence of OW/OB was higher in males (22.2%), than in females (18.8%) (*p* = 0.009).

**Table 1:** Main characteristics of the 21 single nucleotide polymorphisms (SNPs) included in the genetic risk score.

rs code	Nearest gene	Alleles (major/minor)	MAF	*p*	Genotyping success rate	HWE
rs2010899	*AMPD1*	A/C	0.44	0.012	99.8	0.349
rs4135275	*PPARG*	A/G	0.19	0.024	99.9	0.655
rs4912905	*NR3C1*	C/G	0.23	0.004	100.0	0.665
rs7701443	*NR3C1*	A/G	0.40	1.35 × 10^–4^	100.0	0.127
rs13182800	*NR3C1*	C/A	0.24	5.96 × 10^–4^	99.9	0.183
rs9355296	*LPA*	G/A	0.13	0.013	100.0	0.216
rs1524107	*IL-6*	G/A	0.07	0.006	100.0	0.879
rs3211867	*CD36*	C/A	0.07	0.033	100.0	0.728
rs2183013	*CNTFR*	C/G	0.17	0.001	100.0	0.934
rs2515362	*CNTF*	A/G	0.44	0.024	99.9	0.184
rs1800497	*DRD2*	G/A	0.18	0.049	99.8	0.763
rs1019731	*IGF1*	C/A	0.11	0.005	100.0	0.744
rs9939609	*FTO*	T/A	0.40	5.81 × 10^–4^	100.0	0.322
rs4783961	*CETP*	A/G	0.50	0.014	100.0	0.558
rs8068149	*NOS2A*	G/A	0.46	0.010	100.0	0.136
rs7502966	*THRA*	A/G	0.44	0.025	99.7	0.264
rs1568400	*THRA*	A/G	0.26	0.023	100.0	0.349
rs4246444	*FASN*	C/A	0.27	0.008	94.7	0.461
rs1044250	*ANGPTL4*	G/A	0.29	0.005	99.6	0.051
rs17373080	*LXRβ*	G/C	0.32	0.005	99.7	0.523
rs2143511	*PTPN1*	A/G	0.43	0.004	99.9	0.337

Association of SNPs in relation to body mass index (BMI) displayed in p values (*p*).

*AMPD1* (Adenosine Monophosphate Deaminase 1); *ANGPTL4* (Angiopoietin like protein 4); BMI (Boy Mass Index); *CD36* (cluster of differentiation 36); *CETP* (Cholesteryl ester transfer protein); *CNTF* (Ciliary Neurotrophic Factor); *CNTFR* (Ciliary Neurotrophic Factor Receptor); *DRD2* (Dopamine Receptor D2); *FASN* (Fatty acid synthase); *FTO* (Fat mass and obesity-associated gene); HWE (Hardy–Weinberg Equilibrium); *IGF1* (Insulin Like Growth Factor 1); *IL-6* (Interleukin-6); *LPA* (Lipoprotein A); *LXRB* (Liver X receptor beta); MAF (Minor allele frequency); *NOS2A* (Nitric oxide synthase 2); *NR3C1* (Nuclear receptor subfamily 3, group C, member 1); *PPARG* (Peroxisome Proliferator Activated Receptor Gamma); *PTPN1* (Protein Tyrosine Phosphatase Non-Receptor Type 1); *THRA* (Thyroid Hormone Receptor Alpha).

### Associations between SNPs and overweight/obesity: Building and validation of GRS

Initially, 104 SNPs potentially associated with BMI were selected (Supplementary Table 2 and were entered in the multivariate model to build the GRS. From them, we found a final number of 21 SNPs significantly associated with OW/OB in the HELENA study Table 1. Table 2 shows the univariate and multivariate model´s odds ratio (OR) of each of the selected SNPs for the GRS build up. A forest plot is displayed in Fig. 1 to present the OR´s direction (protective/risk) of each SNP. Within our GRS, *AMPD1* rs2010899, *PPARG* rs4135275, *NR3C1* rs4912905, *LPA*rs 9,355,296*, IL-6* rs1524107, *CNTFR* rs2183013, *IGF1* rs1019731, *THRA* rs1568400 and *FASN* rs4246444 had a protective role in the prediction of OW/OB whereas *NR3C1* rs7701443, *NR3C1* rs13182800, *CD36* rs3211867, *CNTF* rs2515362, *DRD2* rs1800497, *FTO* rs9939609, *CETP* rs4783961, *NOS2A* rs8068149, *THRA* rs7502966, *ANGPTL4* rs1044250, *LXRβ* rs17373080 and *PTPN1* rs2143511 increased the risk of OW/OB. Supplementary Fig. 2 shows the calibration curves analysing the correspondence between probabilities of overweight and the real outcome. It can be observed that there is a good agreement between predicted and actual probabilities,thus, the panel of SNPs shows a good adjustment in order to predict OW/OB.

**Table 2:** Association of the 21 single nucleotide polymorphisms (SNPs) of the genetic risk score with OW/OB.

rs code	Chromosome	Univariate*		Multivariate*	
		O.R. (95% C.I.)	*p*	O.R. (95% C.I.)	*p*
rs2010899	1	0.822 (0.665–1.014)	0.068	0.749 (0.596–0.939)	0.012
rs4135275	3	0.738 (0.556–0.967)	0.031	0.709 (0.522–0.951)	0.012
rs4912905	5	0.810 (0.630–1.034)	0.096	0.639 (0.467–0.867)	0.024
rs9355296	6	0.636 (0.448–0.886)	0.009	0.629 (0.430–0.901)	< 0.001
rs1524107	7	0.602 (0.363–0.950)	0.038	0.481 (0.278–0.795)	0.014
rs2183013	9	0.674 (0.495–0.903)	0.010	0.595 (0.425–0.818)	0.033
rs1019731	12	0.681 (0.468–0.967)	0.038	0.571 (0.379–0.837)	0.049
rs1568400	17	0.794 (0.623–1.006)	0.059	0.742 (0.571–0.957)	0.023
rs4246444	17	0.756 (0.592–0.957)	0.022	0.711 (0.548–0.914)	0.009
rs7701443	5	1.170 (0.946–1.447)	0.146	1.919 (1.432–2.579)	0.005
rs13182800	5	1.374 (1.094–1.721)	0.006	1.771 (1.340–2.344)	< 0.001
rs3211867	7	1.487 (1.009–2.159)	0.040	1.576 (1.029–2.384)	0.006
rs2515362	11	1.189 (0.973–1.452)	0.090	1.283 (1.032–1.594)	0.002
rs1800497	11	1.204 (0.924–1.559)	0.162	1.329 (0.998–1.761)	0.024
rs9939609	16	1.496 (1.219–1.838)	< 0.001	1.575 (1.263–1.968)	0.005
rs4783961	16	1.272 (1.034–1.568)	0.023	1.325 (1.058–1.663)	0.014
rs8068149	17	1.190 (0.974–1.456)	0.088	1.331 (1.071–1.656)	0.010
rs7502966	17	1.165 (0.952–1.427)	0.138	1.287 (1.031–1.607)	0.025
rs1044250	19	1.211 (0.976–1.500)	0.079	1.390 (1.100–1.754)	0.006
rs17373080	19	1.313 (1.061–1.624)	0.012	1.388 (1.102–1.748)	0.005
rs2143511	20	1.294 (1.056–1.588)	0.013	1.386 (1.108–1.736)	0.004

SNPs ordered by chromosome number and protector/risk nature of the SNP. *Univariate (individual model of SNP-Overweight/Obesity association) and multivariate model (multiple model SNP-Overweight/Obesity association) displayed with Odds Ratio (O.R.) and 95% confidence interval (C.I.).

**Figure 2:**
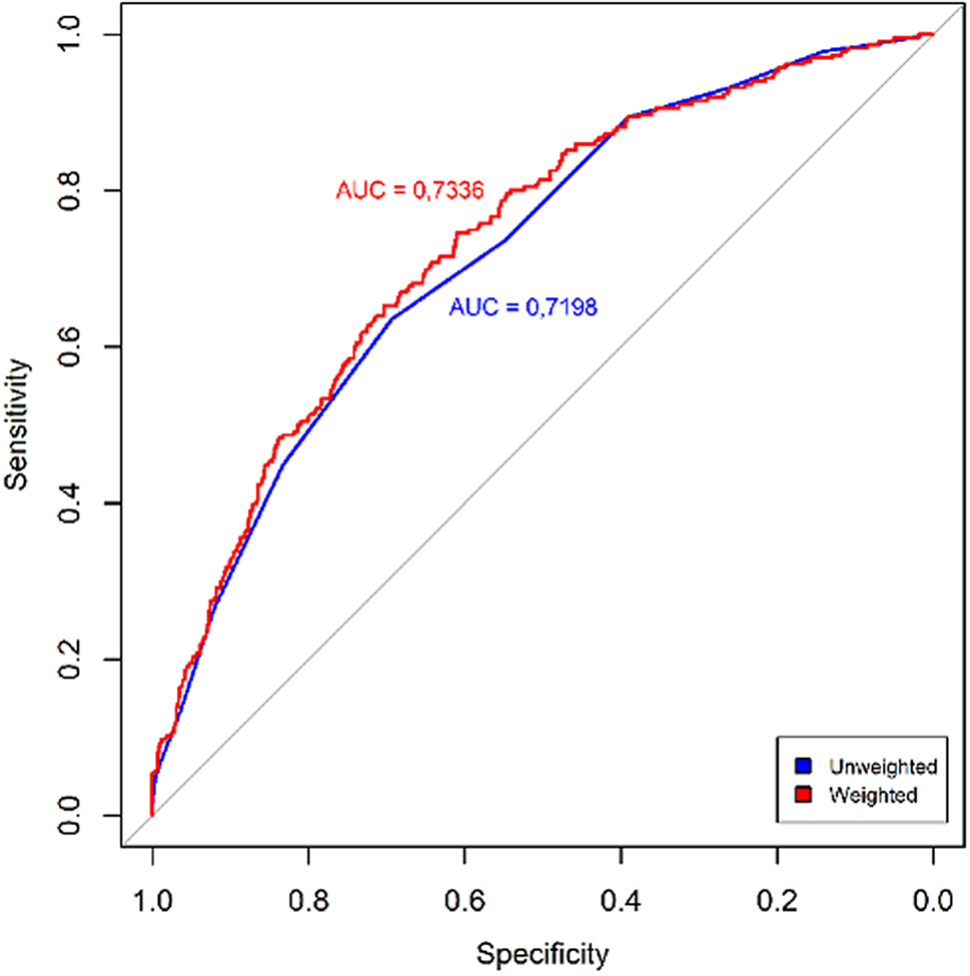
Receiver operating characteristics (ROC) curves of the two genetic risk scores, unweighted (uGRS) and weighted (wGRS). Areas under curves (AUC) are indicated. The straight line represents the ROC expected by chance only.

**Figure 3:**
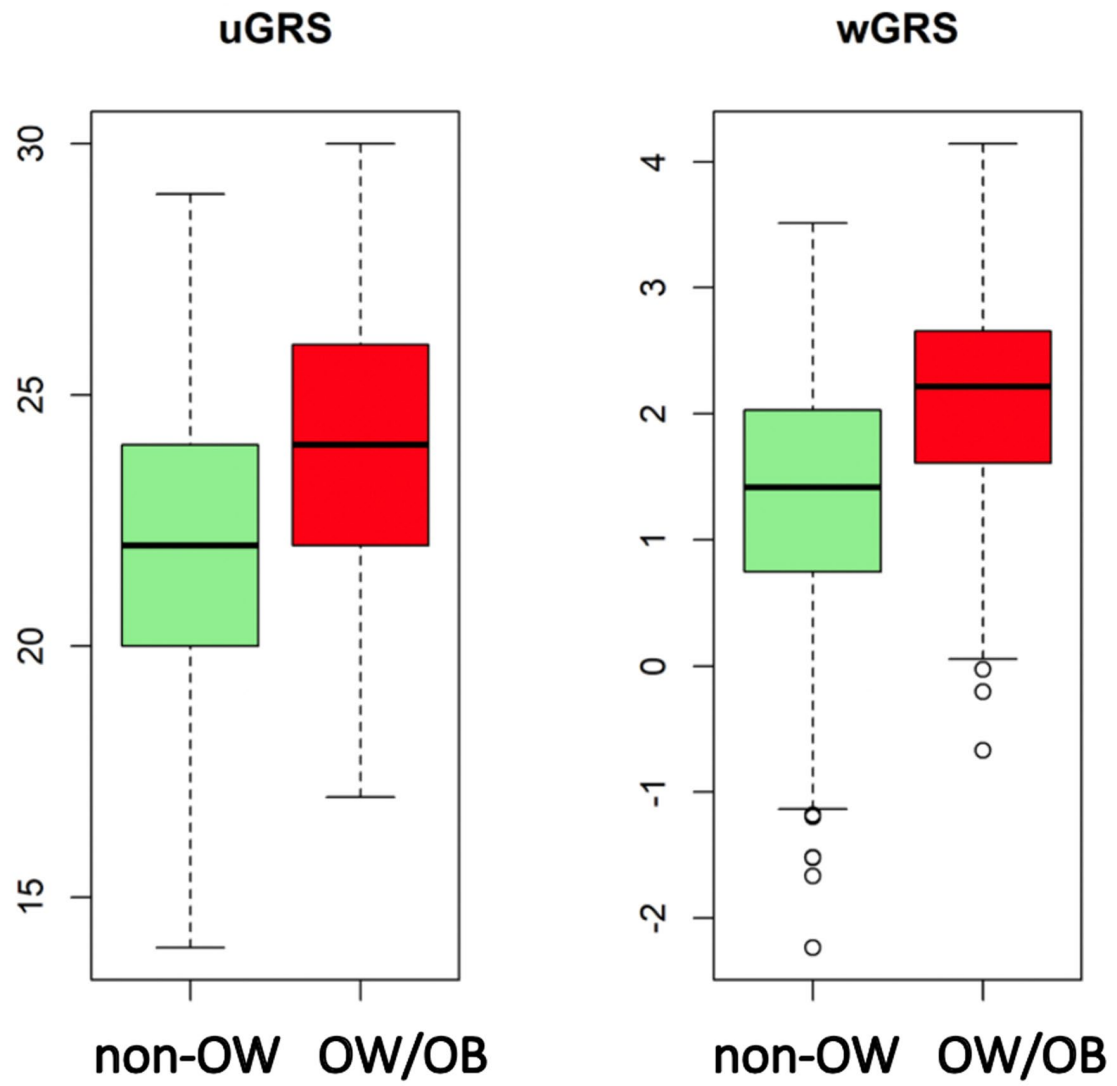
Boxplot of the distribution of unweighted genetic risk score (uGRS) and weighted genetic risk score (wGRS). Legend: Values for the groups of Non-overweight (NON-OW) and Overweight/Obesity (OW/OB).

Using the predictABEL R package^34^, from the multivariate logistic regression model built to predict OW/OB, an uGRS and a wGRS were derived. The predictive ability of the GRS by means of the ROC curve, AUCs and Youden index of uGRS and wGRS models are displayed in Fig. 2. The results of the GRSs´ AUC (uGRS: 0.7198,wGRS: 0.7336) showed a moderate ability to discriminate OW/OB status. AUC´s comparisons displayed statistically significant differences between uGRS and wGRS to predict OW/OB (*p* = 0.043). The discrimination ability of wGRS and uGRS was internally validated by cross-validation using 10 folds. Both GRS provide robust predictions as showed by the AUC results (0.723 and 0.734 for uGRS and wGRS respectively). The distribution of uGRS and wGRS values for the groups of NON-OW and OW/OB by boxplots is displayed in Fig. 3. Both GRS discriminate between groups, but there is no a cut-off point that can clearly separate NON-OW and OW/OB groups. The Youden index was 23.5 for uGRS (specificity 69.4%, sensitivity 63.6%), and − 0.126 for wGRS (specificity 61.1%, sensitivity 74.6%). A more general analysis of sensitivity, specificity, positive and negative predictive value, and accuracy is shown in Table 3.

**Table 3:** Specificity, sensitivity, negative and positive predictive value and accuracy analysis presented in percentages (%).

Cut-off	Spec	Sens	NPV	PPV	Acc	Cut-off	Spec	Sens	NPV	PPV	Acc
**uGRS**						**wGRS**					
30	100	0.42	77.99	100	78.01	4.14	100	0.42	77.99	100	78.01
29	99.88	3.81	78.56	90.00	78.67	3.51	99.88	5.50	78.86	92.85	79.04
28	99.15	6.35	78.89	68.18	78.67	3.30	99.15	8.89	79.34	75.00	79.23
27	96.39	13.55	79.74	51.61	78.11	2.98	96.63	15.25	80.09	56.25	78.67
26	92.07	26.69	81.59	48.83	77.64	2.61	92.07	27.54	81.76	49.61	77.82
25	83.31	44.91	84.22	43.26	74.83	2.26	83.31	48.72	85.15	45.27	75.67
24	69.38	63.55	87.04	37.03	68.10	1.88	69.38	65.25	87.57	37.65	68.47
23	54.74	73.72	88.03	31.57	58.93	1.50	54.74	79.23	90.29	33.15	60.14
22	39.13	89.40	92.87	29.38	50.23	1.10	39.13	89.40	92.87	29.38	50.23
21	26.17	93.22	93.16	26.34	40.97	0.77	26.17	93.22	93.16	26.34	40.97
20	14.04	97.88	95.90	24.39	32.55	0.35	14.04	97.03	94.35	24.23	32.36
19	7.92	98.72	95.65	23.30	27.97	0.03	7.92	98.72	95.65	23.30	27.97
18	2.88	99.57	96.00	22.50	24.22	-0.54	2.88	99.57	96.00	22.50	24.22
17	1.32	100	100	22.30	23.10	-0.82	1.32	100	100	22.30	23.10
16	0.48	100	100	22.15	22.45	-1.20	0.48	100	100	22.15	22.45
15	0.00	100	100	22.09	22.17	-1.67	0.12	100	100	22.09	22.17

Abbreviations: uGRS (Unweighted Genetic Risk Score); wGRS (Weighted Genetic Risk Score); Acc (accuracy); NPV (negative predictive value); PPV (positive predictive value); Sens (sensitivity); Spec (specificity).

### Associations between GRS and adiposity

LRM showed a significant association between GRS and FMI (*p* ≤ 2e−16) in both weighted (β = 0.877) and unweighted (β = 0.312) variants for the total sample.

## Discussion

In the present study, two GRSs, uGRS and wGRS, including 21 SNPs associated with BMI, were successfully developed to assess the risk of overweight and obesity in European adolescents. Hence, to the best of our knowledge, this are the first GRSs with these characteristics in a diversely distributed sample of European adolescents.

There are few previous studies focusing on BMI-specific GRSs with overweight and obesity in European pediatric populations and none exclusively in European adolescents, which reinforces the potential of our GRS analysis. In a cohort of 1142 Finnish preadolescents, Viljakainen et al.^22^ constructed a wGRS to predict the risk of overweight (1.39-fold increased odds) and obesity (1.41-fold increased odds) using 30 BMI-related SNPs, stating that their GRS was poor in predicting short-term longitudinal changes in BMI. In two Finnish children and adolescent cohorts, Seyednasrollah et al.^21^ developed two wGRS of 97 and 19 SNPs previously related to the risk of obesity, and obtained a lightly better prediction accuracy with the 19-SNP GRS than in our study (AUC = 0.769 *vs.* 0.734). However, none of the SNPs used in the two GRSs in European adolescents above mentioned concur with the SNPs utilised to develop our GRS.

The majority of the GRSs developed in pediatric and adolescent populations have been performed in non-European subjects, based on SNPs associated with obesity risk from previous GWAS. Comparatively to the European GRSs, the non-European studies provided a fewer number of selected SNPs related to obesity risk to develop their GRS. In a cross-sectional study of Brazilian children and adolescents (mean age 11.9 ± 2.8 years)^19^, the BMI-specific wGRS composed of 3 SNPs was associated with a 2.65-fold increased risk of overweight and obesity. In a Chinese cohort of children aged 7.3 to 11.1 years, Fang J et al.^35^ developed an uGRS of 11 BMI-related SNPs which explained 0.11 kg/m^2^ BMI increase in those children carrying BMI susceptibility alleles. Lv D et al.^36^ positively associated the cumulative effect of 5 BMI-related SNPs GRS to obesity risk by more than sevenfold increased odds in individuals carrying 5–7 risk alleles (age 11.6 ± 2.5, N = 2977). Finally, SNPs previously related to other cardiometabolic risk factors (hypertension) in a Chinese adolescent population (aged 12.2 ± 3.0 years) were used to develop a 3 SNP GRS positively associated to obesity risk^37^. The increased risk of obesity associated to the mentioned GRSs in non-European subjects seem to have a consistency to some SNPs showed in the present study, despite acknowledging that the subjects origin does not allow to make comparisons in this regard.

The elaboration of a BMI-GRS comprised protector and risk in the same model. Some of those SNPs have been significantly associated with the risk of obesity in previous studies. The A allele of *FTO* rs9939609 polymorphism has been consistently associated with higher BMI and waist circumference in several studies in adults^11^, adolescents^9^ and children^38^. In cohorts of children and adults with European ancestry, Frayling et al.^11^ and Willer et al*.*^39^ found the strongest associations of the A risk allele of *FTO* rs9939609 with BMI. These findings confirm the role of *FTO* rs9939609 in our GRS as risk factor to OW/OB. In a study by Bokor et al.^40^, *CD36* rs3211867 increased the risk of obesity by almost two folds in a cohort of Hungarian obese (N = 307) and normal weight (N = 339) adolescents. Although the study had two independent samples with limited sample size, the findings are consistent with the results of our study. Moreover, in a pooled-study by Solaas et al.^41^, authors observed significant association between *LXRβ* rs17373080 and the risk of T2DM and OW/OB by 1.59-fold increased odds. Equally, in our study, *LXRβ* rs17373080 SNP was associated with a 1.38 fold higher risk of OW/OB. Of note, the last two studies included participants from the HELENA cohort. On the other hand, the present study showed that *THRA rs*1568400 SNP was negatively associated with the risk of OW/OB whereas the same SNP was associated with higher BMI in a cohort of Spanish adults^42^.

Other SNPs included in the GRS developed in the present study have also been associated with obesity-related cardiometabolic risk factors in ethnically diverse adults, but not with overweight or obesity risk. Thus, in a European population, *DRD2 rs*1800497^43^ modified the relationship between birth weight and adulthood educational attainment in Finnish subjects and *FASN rs*4246444^44^ attenuated the effect on low density lipoproteins (LDL) peak particle diameter when consuming a high amount of fat in a Canadian cohort. Within non-European background, in Chinese population, *NR3C1* rs7701443^45^ was significantly associated with a higher risk of metabolic syndrome and CC alleles of *IL-6* rs1524107^46^ had a higher risk of developing nephropathy in T2DM subjects. In addition, *PPARG* rs4135275^47^ was positively associated with glycated hemoglobin and fasting plasma glucose in Taiwanese mental health patients. In pregnant Turkish women, *LPA* rs9355296^48^ was positively related to vascular inflammation as future cardiovascular event indicator. Finally, in a large adult cohort of 5 different ancestry groups, *CETP* rs4783961^49^ was involved in sleep-associated adverse high density lipoproteins (HDL) profiles.

In contrast, as far as we know, several SNPs included our GRSs (i.e., *AMPD1* rs2010899, *NR3C1* rs4912905, *CNTFR* rs2183013, *IGF1* rs1019731 as protector factors and *NR3C1* rs13182800, *CNTF* rs2515362, *NOS2A* rs8068149, *THRA* rs7502966, *ANGPTL4* rs1044250 and *PTPN1* rs2143511 as risk factors) are new predictive factors, as they had not previously been associated with obesity or obesity related diseases nor had been significantly relevant in previous studies.

Additionally, the present GRS was positively tested to evaluate its ability to predict the risk of overweight and obesity in other adiposity estimates (FMI). Previous studies have also identified potential interactions between an obesity-GRS and diet on FMI in English children (9yrs)^50^. Monnereau et al.^51^ constructed 15 SNPs-wGRS related to child BMI in children (6yrs) from Netherlands significantly associated to total fat mass. More so, another study^52^ showed a BMI-based GRS significantly associated to higher body fat mass in Finnish children and adolescents.

Although using the external weight from meta-analyses is the gold standard to build a GRS, when the external weights are not available, the uGRS is commonly used^35,53^. In the present approach, internal weights from the genetic effects of the same study were used. The wGRS outperformed the uGRS in terms of statistical power (0.734 *vs*. 0.723). Conventionally, it is accepted that the AUC in a ROC analysis should be > 0.8 to be of clinical value for screening^54^. When constructing the GRS models, AUC fell short of this threshold combining genetic factors alone. As SNPs themselves have little predictor capacity, we should consider the results obtained to construct the uGRS and wGRS as statistically acceptable, so our wGRS could be replicated in other cohorts with similar characteristics. Thus, our findings add a significant contribution to obesity-specific GRS that may improve the predictive values of obesity biomarkers in adolescents.

Other authors^55^ suggest that traditional predictors, such as family history and childhood obesity have stronger predictive power than models based on the established genetic variants. Nonetheless, the limited predictive ability of genetic variants does not undervalue the role of gene discovery for obesity as, based on the literature, genetic analyses have already provided with promising insights involving BMI regulation^6^. As such, the present GRSs, or future GRS comprising additional SNPs from other genes which do not have (a priori) previous reported associations with obesity, could yield promising results to minimise the risk of cardiovascular events related to obesity.

However, the present study has some limitations. The results should be validated in larger pediatric study populations, also using obesity incidence, in order to test the reliability of this obesity-specific GRS in other populations with similar ethnicity. Despite that some studies on children reporting the prediction of adulthood obesity efficiently 20 to 30 years later^21^, different genetic factors might affect the short-term changes in BMI, especially during periods of rapid growth^56^. Due to the cross-sectional nature of the study, no cause-effect relationship can be determined. Additionally, although the model to develop the GRSs was internally validated performing tenfold cross validation analysis, we understand that the optimal situation would have been an external validation in an independent cohort. Moreover, only selected risk loci are available in the HELENA study. Since the established common variants from GWAS explain a small proportion of the BMI variation^6^, it is likely that other loci from rarer variants, still to be discovered, will emerge when larger sample sizes are included in GWAS. Furthermore, there is no data available regarding the relatedness or the ethnic origins among the studied participants; the allele frequencies and their effect size might be different from non-European populations and the outcome should not be reproduced to other ethnicities. Since the HELENA study selected genes based on candidate genes instead of GWAS, the overlapping effect observed between SNPs of European and non-European adolescents could be possible. More common SNPs in non-European GRSs were found than in European GRSs. This finding could be due to the higher number of GRSs developed in other ethnicities in comparison to the number of GRS performed in European population. Also, we used the same data in the SNPs selection process and in the building model, thus little bias can be produced. Therefore, the results showed in the present study should be considered carefully. Further studies with larger sample size could provide key information of this potential genetic predisposition to obesity. On the other hand, the present study has also some strengths. The multicentric design of HELENA study involved the participation of adolescents from 10 European cities. This allowed the researchers to use a large database with relevant and diverse information from different populations across Europe. Additionally, only few GRSs to predict the overweight and obesity risk have been developed particularly in European adolescents, an understudied population from the early treatment and prevention perspective^21,22^. Similarly to Viljakainen et al.^22^, the proposed genetic score of predisposition to obesity defined in this study might efficiently contribute to discern population at risk for overweight and obesity and not just obesity alone.

In conclusion, our findings suggest that the GRSs developed in the present study (uGRS and wGRS) could be considered as a useful genetic tool to evaluate individual’s predisposition to OW/OB, allowing to advance in the prevention and management of the disease from early stages in life. Future GRS development with larger samples will be able to detect new variants previously not related to obesity that could influence the genetic risk of obesity, other than the common ones.

## Supplementary Information

Supplementary Information 1.

Supplementary Information 2.

Supplementary Information 3.
