# Supplementary figures and images for: Development of a Genetic Risk Score to predict the risk of overweight and obesity in European adolescents from the HELENA study
Source: Sci Rep. 2021 Feb 4;11:3067. doi: 10.1038/s41598-021-82712-4 (PMC7862459; doi:10.1038/s41598-021-82712-4)

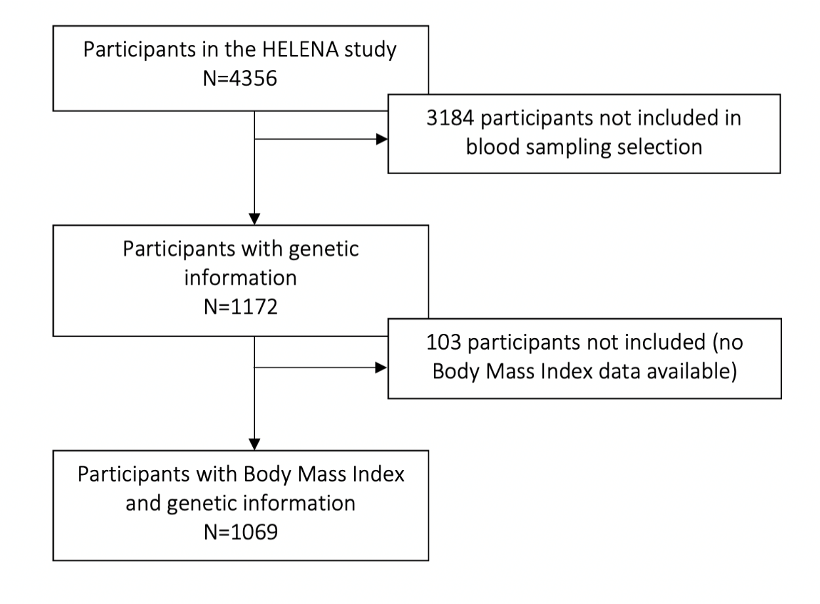

Supplement: Supplementary file 1 — Supplementary Information 1. [file 41598_2021_82712_MOESM1_ESM.tiff]

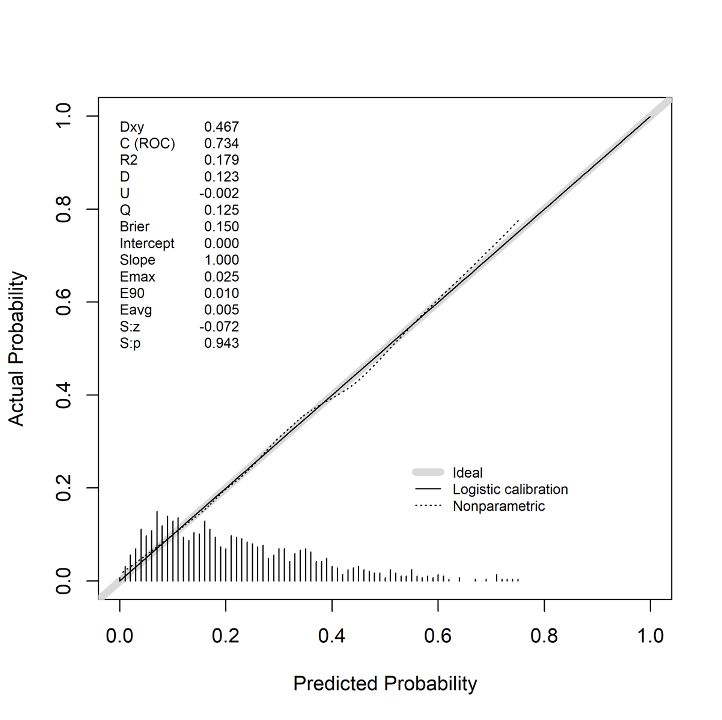

Supplement: Supplementary file 2 — Supplementary Information 2. [file 41598_2021_82712_MOESM2_ESM.tiff]
