# Supplementary material for: Development of a Genetic Risk Score to predict the risk of overweight and obesity in European adolescents from the HELENA study
Source: Sci Rep. 2021 Feb 4;11:3067. doi: 10.1038/s41598-021-82712-4 (PMC7862459; doi:10.1038/s41598-021-82712-4)
Supplement: Supplementary file 3 — Supplementary Information 3. [file 41598_2021_82712_MOESM3_ESM.docx]

**Development of a Genetic Risk Score to predict the risk of overweight and obesity in European adolescents from the HELENA study.**

**Miguel Seral-Cortes^1,2*^, Sergio Sabroso-Lasa^3^, Pilar De Miguel-Etayo^1,2,4^,** **Marcela Gonzalez-Gross^4,5^**, **Eva Gesteiro^5^, Cristina Molina-Hidalgo^6^,** **Stefaan De Henauw^7^,** **Frederic Gottrand^8^** ,**Christina Mavrogianni^9^, Yannis Manios^10^, Maria Plada^11^**, **Kurt Widhalm^12^, Antonios Kafatos^13^,** **Éva Erhardt^14^,** **Aline Meirhaeghe^15^, Diego Salazar-Tortosa^16^, Jonatan Ruiz^17^, Luis Moreno^1,2,4^, Luis Mariano Esteban^18^ and Idoia Labayen^19^.**

1: Growth, Exercise, NUtrition and Development (GENUD) Research Group. Instituto Agroalimentario de Aragón (IA2), Instituto de Investigación Sanitaria Aragón (IIS Aragón). Universidad de Zaragoza, Zaragoza, Spain.

2: Departmento de Fisiatría y Enfermería, Facultad de Ciencias de la Salud, Universidad de Zaragoza, Spain.

3: Instituto de Investigación Sanitaria Aragón. Zaragoza, Spain.

4: CIBER Fisiopatología de la Obesidad y Nutrición (CIBERobn), Instituto de Salud Carlos III, Madrid, Spain.

5: ImFine Research Group, Department of Health and Human Performance, Facultad de Ciencias de la Actividad Física y del Deporte-INEF, Universidad Politécnica de Madrid, Madrid, Spain.

6: EFFECTS 262 Department of Medical Physiology, School of Medicine, University of Granada, 18071 Granada, Spain.

7: Department of Public Health, Faculty of Medicine and Health Sciences, Ghent University, Belgium.

8: Faculty of Medicine, University Lille, Lille, France

9: Department of Nutrition and Dietetics, Harokopio University, Athens, Greece

10: Department of Nutrition and Dietetics, School of Health Science & Education, Harokopio University, Athens, Greece

11: University of Crete School of Medicine, Crete, Greece.

12: Div. Gastroenterology and Hepatology , Dept. Internal Med III;  Med Univ. Vienna, Austria and Austrian Academic Institute for Clinical Nutrition, Vienna, Austria.

13: Faculty of Medicine, University of Crete, Crete, Greece.

14: Department of Pediatrics, Medical School, University of Pécs, Pécs, Hungary

15: UMR1167, RID-AGE, Risk Factors and Molecular Determinants of Aging-Related Diseases, Centre Hosp. Univ Lille, Institut Pasteur de Lille, Université de Lille, Lille, France.

16: Department of Ecology and Evolutionary Biology, University of Arizona, AZ, USA.

17: Departmento de Actividad Física y Deporte, Faculty of Sport Sciences, Universidad de Granada, Granada, Spain

18: Escuela Politécnica de La Almunia. Universidad de Zaragoza, Zaragoza, Spain.

19: Departmento de Ciencias de la Salud, Universidad Pública de Navarra, Pamplona, Spain.

* Corresponding author: Miguel Seral-Cortes

GENUD (Growth, Exercise, NUtrition and Development) Research Group

Faculty of Health Sciences, Universidad de Zaragoza

C/ Domingo Miral s/n, 50009 Zaragoza, Spain.

E-mail address: [mseral@unizar.es](mailto:mseral@unizar.es)

List of emails:

[ssabroso@iisaragon.es](mailto:ssabroso@iisaragon.es); [pilardm@unizar.es](mailto:pilardm@unizar.es); [marcela.gonzalez.gross@upm.es](mailto:marcela.gonzalez.gross@upm.es); [eva.gesteiro@upm.es](mailto:eva.gesteiro@upm.es); [criismh@correo.ugr.es](mailto:criismh@correo.ugr.es); [Stefaan.DeHenauw@UGent.be](mailto:Stefaan.DeHenauw@UGent.be); [Frederic.GOTTRAND@CHRU-LILLE.FR](mailto:Frederic.GOTTRAND@CHRU-LILLE.FR); cmavrog@hua.gr; [manios@hua.gr](mailto:manios@hua.gr); mplada@hotmail.com;

[kurt.widhalm@meduniwien.ac.at](mailto:kurt.widhalm@meduniwien.ac.at); [kafatos@med.uoc.gr](mailto:kafatos@med.uoc.gr); [erhardt.eva@pte.hu](mailto:erhardt.eva@pte.hu); [aline.meirhaeghe@pasteur-lille.fr](mailto:aline.meirhaeghe@pasteur-lille.fr); [ruizj@ugr.es](mailto:ruizj@ugr.es); [dsalazar@ugr.es](mailto:dsalazar@ugr.es); [lmoreno@unizar.es](mailto:lmoreno@unizar.es); [lmeste@unizar.es](mailto:lmeste@unizar.es) and idoia.labayen@unavarra.es

**Supplemental Material**

**Supplemental Material. Figure 1.** Diagram flow chart of sample selection process.

**Supplemental Material Table 1.** Characteristics of study participants meeting inclusion criteria in the HELENA study.

|  | **Total** | **Males** | **Females** | *p* |
| --- | --- | --- | --- | --- |
|  | n =1069 | n =520 | n =549 |  |
| Age(yrs.) | 14.6  (13.5-15.7) | 14.6  (13.6-15.7) | 14.6  (13.5-15. 7) | 0.722 |
| Body Weight (kg) | 56.7  (49.7-64.5) | 60.0  (51.8-69.2) | 54.5  (48.3-60.5) | <0.001 |
| Height (cm) | 164.5  (158.1-171.5) | 169.85  (161.4-176.1) | 161.60 (156.4-166.0) | <0.001 |
| BMI categories, (N, %) |  |  |  | 0.009 |
| Normal | 833 (74.4) | 387 (77.7) | 446 (81.2) |  |
| Overweight/Obesity | 236 (25.5) | 133 (22.2) | 103 (18.8 |  |

Unless otherwise stated, the values are presented as median (p25 – p75). Mann-Whitney-Wilcoxon test was performed to observe differences between sex in Age, Body Weight and Height. Chi-square test was performed to observe differences in sex between Normal and Overweight/Obese categories respectively.

**Supplemental Material. Table 2**: Selection of 104 candidate single nucleotide polymorphisms (SNPs) to build the genetic risk score (GRS).

| **SNP code** | **Gene** | **HWE** | **p-value univariate** | **p-value multivariate** |
| --- | --- | --- | --- | --- |
| rs17037390 | *MTHFR* | 0.069 | 0.196 | ns |
| rs9651118 | *MTHFR* | 0.354 | 0.047 | ns |
| rs4927193 | *PCSK9* | 0.426 | 0.036 | 0.061 |
| rs499718 | *PCSK9* | 0.343 | 0.101 | ns |
| rs2010899 | *AMPD1* | 0.349 | 0.081 | 0.074 |
| rs2774276 | *USF1* | 0.183 | 0.116 | ns |
| rs12733285 | *ADIPOR1* | 0.458 | 0.174 | ns |
| rs1518110 | *IL10* | 0.950 | 0.149 | ns |
| rs3024490 | *IL10* | 0.891 | 0.187 | ns |
| rs1800872 | *IL10* | 0.955 | 0.171 | ns |
| rs1800871 | *IL10* | 0.988 | 0.164 | ns |
| rs934778 | *POMC* | 0.563 | 0.087 | ns |
| rs35683 | *GHRL* | 0.934 | 0.032 | ns |
| rs2075356 | *GHRL* | 0.108 | 0.134 | ns |
| rs12490265 | *PPARG* | 0.316 | 0.089 | ns |
| rs4135247 | *PPARG* | 0.961 | 0.030 | ns |
| rs2921188 | *PPARG* | 0.977 | 0.121 | ns |
| rs4135275 | *PPARG* | 0.655 | 0.008 | 0.058 |
| rs361072 | *PIK3CB* | 0.729 | 0.064 | ns |
| rs16861210 | *ADIPOQ* | 0.997 | 0.148 | ns |
| rs822396 | *ADIPOQ* | 0.059 | 0.175 | ns |
| rs2970847 | *PPARGC1A* | 0.934 | 0.173 | 0.036 |
| rs12502572 | *UCP1* | 0.568 | 0.109 | 0.035 |
| rs7688743 | *UCP1* | 0.988 | 0.157 | ns |
| rs10482682 | *NR3C1* | 0.884 | 0.043 | ns |
| rs10482655 | *NR3C1* | 0.647 | 0.157 | ns |
| rs4912905 | *NR3C1* | 0.665 | 0.066 | 0.005 |
| rs2963151 | *NR3C1* | 0.981 | 0.031 | ns |
| rs2963155 | *NR3C1* | 0.438 | 0.004 | ns |
| rs7701443 | *NR3C1* | 0.127 | 0.158 | 0.014 |
| rs4244032 | *NR3C1* | 0.274 | 0.004 | ns |
| rs13182800 | *NR3C1* | 0.183 | 0.004 | ns |
| rs1800562 | *HFE* | 0.624 | 0.118 | 0.071 |
| rs2076684 | *PLAGL1* | 0.081 | 0.169 | ns |
| rs11751605 | *LPA* | 0.968 | 0.034 | 0.028 |
| rs10755578 | *LPA* | 0.249 | 0.036 | ns |
| rs9355296 | *LPA* | 0.216 | 0.009 | 0.124 |
| rs1524107 | *IL6* | 0.879 | 0.064 | <0.001 |
| rs16143 | *NPY* | 0.232 | 0.073 | ns |
| rs1527479 | *CD36* | 0.344 | 0.050 | ns |
| rs3211867 | *CD36* | 0.728 | 0.011 | ns |
| rs3211908 | *CD36* | 0.724 | 0.067 | ns |
| rs2183013 | *CNTFR* | 0.934 | 0.002 | 0.005 |
| rs12554080 | *CNTFR* | 0.484 | 0.031 | ns |
| rs12551429 | *CNTFR* | 0.937 | 0.008 | 0.089 |
| rs6476454 | *CNTFR* | 0.084 | 0.191 | ns |
| rs1080750 | *CNTFR* | 0.093 | 0.056 | ns |
| rs2246293 | *ABCA1* | 0.760 | 0.057 | 0.195 |
| rs2282310 | *GPRL10* | 0.769 | 0.172 | ns |
| rs1711869 | *PRLHR* | 0.814 | 0.054 | ns |
| rs8192524 | *PRLHR* | 0.839 | 0.074 | 0.249 |
| rs3213225 | *IGF2* | 0.995 | 0.140 | ns |
| rs3213223 | *IGF2* | 0.907 | 0.018 | ns |
| rs3213221 | *IGF2* | 0.904 | 0.159 | ns |
| rs214083 | *NUCB2* | 0.299 | 0.053 | ns |
| rs7127347 | *NUCB2* | 0.359 | 0.114 | ns |
| rs11030101 | *BDNF* | 0.416 | 0.122 | ns |
| rs2509914 | *CNTF* | 0.272 | 0.119 | ns |
| rs2515363 | *CNTF* | 0.190 | 0.101 | ns |
| rs2515362 | *CNTF* | 0.184 | 0.092 | ns |
| rs7930460 | *UCP3* | 0.761 | 0.192 | ns |
| rs1800497 | *ANKK1* | 0.763 | 0.076 | ns |
| rs10891549 | *DRD2* | 0.951 | 0.107 | 0.009 |
| rs2242592 | *DRD2* | 0.806 | 0.020 | 0.066 |
| rs2734838 | *DRD2* | 0.860 | 0.028 | 0.123 |
| rs2005313 | *DRD2* | 0.274 | 0.003 | 0.047 |
| rs683271 | *NNMT* | 0.876 | 0.026 | ns |
| rs2301128 | *NNMT* | 0.144 | 0.163 | 0.040 |
| rs2058035 | *ADIPOR2* | 0.514 | 0.099 | ns |
| rs7975600 | *ADIPOR2* | 0.902 | 0.076 | ns |
| rs1044471 | *ADIPOR2* | 0.073 | 0.066 | ns |
| rs1520220 | *IGF1* | 0.909 | 0.124 | ns |
| rs1019731 | *IGF1* | 0.744 | 0.028 | 0.164 |
| rs2162679 | *IGF1* | 0.632 | 0.107 | ns |
| rs6082 | *LIPC* | 0.491 | 0.183 | ns |
| rs9939609 | *FTO* | 0.322 | <0.001 | <0.001 |
| rs4783961 | *CETP* | 0.558 | 0.031 | 0.076 |
| rs5923 | *LCAT* | 0.584 | 0.067 | ns |
| rs8068149 | *NOS2* | 0.136 | 0.100 | 0.002 |
| rs12944039 | *NOSA2* | 0.225 | 0.120 | 0.062 |
| rs7502966 | *THRA* | 0.264 | 0.195 | 0.131 |
| rs1568400 | *THRA* | 0.349 | 0.071 | 0.026 |
| rs16940439 | *PYY* | 0.572 | 0.173 | 0.045 |
| rs1859223 | *PYY* | 0.433 | 0.153 | 0.186 |
| rs2075559 | *COL1A1* | 0.513 | 0.096 | ns |
| rs2075558 | *COL1A1* | 0.360 | 0.163 | ns |
| rs12949488 | *NA* | 0.845 | 0.186 | ns |
| rs4246444 | *FASN* | 0.461 | 0.032 | 0.024 |
| rs4485435 | *FASN* | 0.338 | 0.118 | ns |
| rs2241393 | *C3* | 0.050 | 0.071 | 0.030 |
| rs344541 | *C3* | 0.986 | 0.086 | ns |
| rs2230205 | *C3* | 0.464 | 0.063 | 0.016 |
| rs3219178 | *RETN* | 0.569 | 0.090 | ns |
| rs3745369 | *RETN* | 0.431 | 0.196 | ns |
| rs1044250 | *ANGPTL4* | 0.051 | 0.068 | ns |
| rs17373080 | *NR1H2* | 0.523 | 0.003 | ns |
| rs2695121 | *NR1H2* | 0.860 | 0.025 | ns |
| rs3219281 | *POLD1* | 0.149 | 0.013 | 0.087 |
| rs553359 | *PLTP* | 0.363 | 0.102 | ns |
| rs6067472 | *PTPN1* | 0.688 | 0.039 | ns |
| rs2143511 | *PTPN1* | 0.337 | 0.012 | 0.020 |
| rs6020608 | *PTPN1* | 0.672 | 0.135 | ns |
| rs968701 | *PTPN1* | 0.578 | 0.074 | ns |
| rs738504 | *TFR2* | 0.297 | 0.116 | ns |

Legend: SNP code, nearest gene of located SNP, Hardy Weinberg Equilibrium (HWE) and p-value of SNPs in univariate and multivariate analysis with p <0.20 threshold displayed. In the multivariate column, p-value displayed as *ns* if SNP p >0.20.

**Supplemental Material Figure 2.** Calibration Curve. Correspondence between probabilities of OW/OB (predicted probability) and real outcome (actual probability).

Legend: Ideal, Logistic calibration and Nonparametric lines are displayed.

**Appendix**

HELENA Study Group.

Co-ordinator: Luis A. Moreno.

Core Group members: Luis A. Moreno, Fréderic Gottrand, Stefaan De Henauw, Marcela González-Gross, Chantal Gilbert.

Steering Committee: Anthony Kafatos (President), Luis A. Moreno, Christian Libersa, Stefaan De Henauw, Sara Castelló, Fréderic Gottrand, Mathilde Kersting, Michael Sjöstrom, Dénes Molnár, Marcela González-Gross, Jean Dallongeville, Chantal Gilbert, Gunnar Hall, Lea Maes, Luca Scalfi.

Project Manager: Pilar Meléndez.

1.Universidad de Zaragoza (Spain)

Luis A. Moreno, Jose A. Casajús, Jesús Fleta, Gerardo Rodríguez, Concepción Tomás, María I. Mesana, Germán Vicente-Rodríguez, Adoración Villarroya, Carlos M. Gil, Ignacio Ara, Juan Fernández Alvira, Gloria Bueno, Olga Bueno, Juan F. León, Jesús Mª Garagorri, Idoia Labayen, Iris Iglesia, Silvia Bel, Luis A. Gracia Marco, Theodora Mouratidou, Alba Santaliestra-Pasías, Iris Iglesia, Esther González-Gil, Pilar De Miguel-Etayo, Cristina Julián, Mary Miguel-Berges, Isabel Iguacel, Azahara Ruperez and Miguel Seral-Cortes.

2. Consejo Superior de Investigaciones Científicas (Spain)

Ascensión Marcos, Julia Wärnberg, Esther Nova, Sonia Gómez, Ligia Esperanza Díaz, Javier Romeo, Ana Veses, Belén Zapatera, Tamara Pozo, David Martínez.

3. Université de Lille 2 (France).

Laurent Beghin, Christian Libersa, Frédéric Gottrand, Catalina Iliescu, Juliana Von Berlepsch.

4. Research Institute of Child Nutrition Dortmund, Rheinische Friedrich-Wilhelms-Universität Bonn (Germany)

Mathilde Kersting, Wolfgang Sichert-Hellert, Ellen Koeppen.

5. Pécsi Tudományegyetem (University of Pécs) (Hungary) Dénes Molnar, Eva Erhardt, Katalin Csernus, Katalin Török, Szilvia Bokor, Mrs. Angster, Enikö Nagy, Orsolya Kovács, Judit Répasi.

6. University of Crete School of Medicine (Greece)

Anthony Kafatos, Caroline Codrington, María Plada, Angeliki Papadaki, Katerina Sarri, Anna Viskadourou, Christos Hatzis, Michael Kiriakakis, George Tsibinos, Constantine Vardavas, Manolis Sbokos, Eva Protoyeraki, Maria Fasoulaki.

7. Institut für Ernährungs- und Lebensmittelwissenschaften – Ernährungphysiologie. Rheinische Friedrich Wilhelms Universität (Germany)

Peter Stehle, Klaus Pietrzik, Marcela González-Gross, Christina Breidenassel, Andre Spinneker, Jasmin Al-Tahan, Miriam Segoviano, Anke Berchtold, Christine Bierschbach, Erika Blatzheim, Adelheid Schuch, Petra Pickert.

8.University of Granada (Spain)

Manuel J. Castillo, Ángel Gutiérrez, Francisco B Ortega, Jonatan R Ruiz, Enrique G Artero, Vanesa España, David Jiménez-Pavón, Palma Chillón, Cristóbal Sánchez-Muñoz, Magdalena Cuenca.

9. Istituto Nazionalen di Ricerca per gli Alimenti e la Nutrizione (Italy)

Davide Arcella, Elena Azzini, Emma Barrison, Noemi Bevilacqua, Pasquale Buonocore, Giovina Catasta, Laura Censi, Donatella Ciarapica, Paola D'Acapito, Marika Ferrari, Myriam Galfo, Cinzia Le Donne, Catherine Leclercq, Giuseppe Maiani, Beatrice Mauro, Lorenza Mistura, Antonella Pasquali, Raffaela Piccinelli, Angela Polito, Romana Roccaldo, Raffaella Spada, Stefania Sette, Maria Zaccaria. 10. University of Napoli "Federico II" Dept of Food Science (Italy)

Luca Scalfi, Paola Vitaglione, Concetta Montagnese.

11. Ghent University (Belgium)

Ilse De Bourdeaudhuij, Stefaan De Henauw, Tineke De Vriendt, Lea Maes, Christophe Matthys, Carine Vereecken, Mieke de Maeyer, Charlene Ottevaere, Inge Huybrechts.

12. Medical University of Vienna (Austria)

Kurt Widhalm, Katharina Phillipp, Sabine Dietrich, Birgit Kubelka
Marion Boriss-Riedl.

13. Harokopio University (Greece)

Yannis Manios, Eva Grammatikaki, Zoi Bouloubasi, Tina Louisa Cook, Sofia Eleutheriou, Orsalia Consta, George Moschonis, Ioanna Katsaroli, George Kraniou, Stalo Papoutsou, Despoina Keke, Ioanna Petraki, Elena Bellou, Sofia Tanagra, Kostalenia Kallianoti, Dionysia Argyropoulou, Stamatoula Tsikrika, Christos Karaiskos.

14. Institut Pasteur de Lille (France)

Jean Dallongeville, Aline Meirhaeghe.

15. Karolinska Institutet (Sweden) Michael Sjöstrom, Jonatan R Ruiz, Francisco B. Ortega, María Hagströmer, Anita Hurtig Wennlöf, Lena Hallström, Emma Patterson, Lydia Kwak, Julia Wärnberg, Nico Rizzo.

16. Asociación de Investigación de la Industria Agroalimentaria (Spain) Jackie Sánchez-Molero, Sara Castelló, Elena Picó, Maite Navarro, Blanca Viadel, José Enrique Carreres, Gema Merino, Rosa Sanjuán, María Lorente, María José Sánchez.

17. Campden BRI (United Kingdom)

Chantal Gilbert, Sarah Thomas, Elaine Allchurch, Peter Burgess.

18. SIK - Institutet foer Livsmedel och Bioteknik (Sweden)

Gunnar Hall, Annika Astrom, Anna Sverkén, Agneta Broberg.

19. Meurice Recherche & Development asbl (Belgium)

Annick Masson, Claire Lehoux, Pascal Brabant, Philippe Pate, Laurence Fontaine. 20. Campden & Chorleywood Food Development Institute (Hungary)

Andras Sebok, Tunde Kuti, Adrienn Hegyi.

21. Productos Aditivos SA (Spain)

Cristina Maldonado, Ana Llorente.

22. Cárnicas Serrano SL (Spain)

Emilio García.

23. Cederroth International AB (Sweden)

Holger von Fircks, Marianne Lilja Hallberg, Maria Messerer.

24. Lantmännen Food R&D (Sweden)

Mats Larsson, Helena Fredriksson, Viola Adamsson, Ingmar Börjesson.

25. European Food Information Council (Belgium)

Laura Fernández, Laura Smillie, Josephine Wills.

26.Universidad Politécnica de Madrid (Spain)

Marcela González-Gross, Raquel Pedrero-Chamizo, Agustín Meléndez, Jara Valtueña, David Jiménez-Pavón, Ulrike Albers, Pedro J. Benito, Juan José Gómez Lorente, David Cañada, Alejandro Urzanqui, Rosa María Torres, Paloma Navarro.
